# Environmentally friendly Au@CNC hybrid systems as prospective humidity sensors†

**DOI:** 10.1039/d0ra07300h

**Published:** 2020-09-21

**Authors:** Maria S. Koroleva, Chantal Tracey, Yuri A. Sidunets, Mikhail A. Torlopov, Vasily I. Mikhaylov, Pavel V. Krivoshapkin, Ilia S. Martakov, Elena F. Krivoshapkina

**Affiliations:** Institute of Chemistry of Federal Research Centre “Komi Science Centre of the Ural Branch of the Russian Academy of Sciences” 48 Pervomayskaya Street 167000 Syktyvkar Russia gmartakov@gmail.com kef@scamt-itmo.ru; ITMO University Lomonosova Str., 9 Saint Petersburg 191002 Russian Federation

## Abstract

Both cellulose nanocrystals and gold nanoparticles show immense potential for biological and chemical applications. Gold nanoparticles, which tend to aggregate, are hybridized with cellulose nanocrystals to form stable inorganic–organic hybrids in which nanocellulose acts as a green supporting material for the catalytically active gold nanoparticles. A green synthesis approach was taken, and hydrothermal treatment was used to reduce electrostatic repulsion between the gold nanoparticles and the cellulose nanocrystals to promote heteroaggregation instead of homoaggregation. AFM analysis showed hybrid films to be hygroscopic, suggesting that they would respond to changes in humidity. Laser diffraction and fluorescence quenching were used to determine how hybrid films respond to changes in humidity. Hybrid films were found to respond to changes in humidity quickly, reversibly, and autonomously, making them ideal for use as or in a humidity sensor. Gold nanoparticles were shown to enhance the hybrid response to ambient moisture, causing them to show a linear dependence on changes in humidity, making the hybrid controllable, highly sensitive, and a viable prospective material for humidity sensing applications.

## Abstract 2

Cellulose nanocrystals are a “green” support for nanogold. Hybrid films quickly and autonomously respond to changes in humidity and can be used in sensing applications.

## Graphical abstract

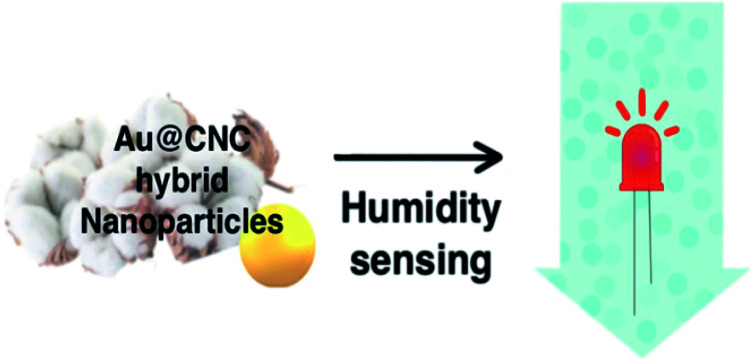

## 1. Introduction

Advanced materials designed with green chemistry in mind tend to be safe, biocompatible and nontoxic.^1,2^ One way to guarantee the manifestation of these properties is by using starting materials with the desired characteristics to make the advanced material. Polysaccharides are well-known for their safety, biocompatibility and non-toxicity, and have been used as a fundamental material in human-related applications for a long time.^3–5^ By downsizing polysaccharide-based materials to the nanoscale, they have high specific surface area, high dispersibility, and high stability,^6,7^ making them viable catalyst carriers,^8^ coatings,^9^ membranes,^10^ and drug delivery systems^11,12^ – among other uses. Bulk and microcrystalline polysaccharide-based materials lack these advantages. Hence, polysaccharide-based nanocrystals – chitin and cellulose in particular – are widely used and are extremely promising when it comes to potential applications.

Due to their unique properties and applications in medicine,^13^ optoelectronics,^14^ and as sensors,^15,16^ gold nanoparticles (AuNPs) have attracted tantamount attention in the past few decades. AuNPs are one of the most effective catalysts for a number of important chemical reactions.^17^ Unfortunately, due to the low repulsive forces between particles resulting in a tendency to agglomerate in solution, colloidal gold is generally unstable, leading to the formation of large particles with reduced catalytic activity. It is therefore usually encapsulated in polymers or supported by more colloidally stable materials (*e.g.* metal/silica oxides, carbon nanomaterials *etc.*) in an effort to reduce homoaggregation and maintain its effectiveness. As science and technology shift towards more environmentally friendly and sustainable resources and processes, so have the means of improving colloidal gold stability. There has been an increase in supports with low or no environmental impact. Polysaccharides, especially cellulose nanocrystals, have emerged as one of the more ideal candidates for “green” supporting materials for not only colloidal gold, but noble metal nanoparticles when it comes to the fabrication of inorganic–organic nanohybrids.

The deposition of AuNPs on the surface of cellulose nanocrystals opens up a new approach to hybrid heterogeneous catalysts for organic transformations. Be that as it may, to deposit AuNPs on cellulose surfaces it is necessary to either modify one of the components to promote covalent bonding or to “tune” its surface charge to generate electrostatic attraction. Either method causes heteroaggregation instead of homoaggregation. To do this, we propose the hydrothermal treatment of mixed gold and cellulose nanocrystals sols to form gold–cellulose nanohybrids. In this paper, we show potential applications of these hybrid nanoparticles in humidity sensing using different approaches.

## 2. Experimental section

### 2.1. Materials and methods

#### 2.1.1 Reagents

Cotton microcrystalline cellulose (>99.8% Glc), copper(ii) acetate monohydrate ((CH_3_COO)_2_Cu·H_2_O, ≥99.0%), rhodamine B for fluorescence, trisodium citrate dihydrate (Na_3_C_6_H_5_O_7_·2H_2_O, ≥99.0%) were purchased from Sigma-Aldrich. Hydrogen peroxide (H_2_O_2_, 30%) and concentrated nitric acid (HNO_3_, 65%) were purchased from LenReactiv (Russia). Trilon B was obtained from NevaReactiv (Russia), and glacial acetic acid was purchased from Vekton (Russia). Hydrogen chloride (HCl, ≥99.0%) was purchased from Sigma-Tech (Russia). The water (DI) used in the syntheses and experiments was ultrapure (Millipore, 18.2 MΩ cm).

#### 2.1.2 Preparation methods and treatment conditions

Cellulose nanocrystals (CNCs) were prepared by hydrolysing cotton microcrystalline cellulose with glacial acetic acid in the presence of (CH_3_COO)_2_Cu and H_2_O_2_. Briefly, a (CH_3_COO)_2_Cu (2.04 mmol, 0.405 g) and CH_3_COOH (100 cm^3^) mixture was stirred vigorously while being heated. Upon boiling, microcrystalline cellulose (10 g) was added to the solution. The mixture was kept under reflux and vigorous stirring for 40 min. 30% H_2_O_2_ solution (1 cm^3^) was then added every 5 minutes for one hour. After the hour, the mixture was allowed to cool, diluted with deionized water (in a ratio of 1 : 3) and stirred for 10 min. The precipitated solution was centrifugated at 4000 rpms for 7 min. The resulting solution was homogenized and neutralized (pH 7). Trilon B (1.85 mmol, 0.688 g) was added to the homogenized mixture to remove excess copper ions. The mixture was stirred for 10 min then allowed to sediment overnight. The supernatant containing the copper ions was removed *via* centrifugation at 4000 rpms for 15 min. This was repeated until the blue supernatant was fully removed from the mixture. Afterwards, the CNC slurry was diluted with deionized water (20 cm^3^) and homogenized. Stable CNC hydrosols were isolated by centrifuging the homogeneous mixture at 4000 rpms for 30 min then subjecting it to 3 days of dispersion dialysis.^18^

The CNCs were treated under hydrothermal conditions to determine optimal treatment conditions. Hydrothermal treatment entailed filling a PTFE autoclave to 80% of its total volume and heating in an oven for 12 hours. CNC concentration was determined to be 0.0021 g cm^−3^. The zeta-potential, apparent size of the particles, and pH value were measured both prior to and after hydrothermal treatment.

Chloroauric acid was synthesised *via* a direct preparation from pure gold foil. Gold (0.0739 g) was measured into a 50 cm^3^ one-neck round bottom flask. Concentrated HCl (1 cm^3^) and 3 drops of concentrated HNO_3_ were added to the flask before sealing. The sealed flask was heated under stirring until the solution boiled. After this, the flask was opened and deionized water (1 cm^3^) was added to remove excess acid while ensuring the gold remained completely dissolved. Hereafter, the water solutions of chloroauric acid was diluted up to volume 25 cm^3^ to obtain 0.015 M solution.

Gold nanoparticles (AuNPs) were synthesised using the citrate method,^19^ with chloroauric acid (HAuCl_4_) and sodium citrate (Na_3_C_6_H_5_O_7_·2H_2_O) as precursors. Aqueous HAuCl_4_ (1 mmol dm^−3^; 35 cm^3^) was boiled while being slowly stirred (300 rpm) to ensure even heating. Aqueous Na_3_C_6_H_5_O_7_·2H_2_O (34 mmol dm^−3^; 1.75 cm^3^) was then added to the boiling solution, and the stirring increased (1000 rpm). A gradual colour change from yellow to blue to wine red was observed. The mixture was boiled for another 15 minutes under stirring and the resulting sol was allowed to cool to room temperature.

The gold nanoparticle–cellulose nanocrystal (Au@CNC) hybrids were synthesized by mixing the sols and then subjecting the hybrid sol to hydrothermal treatment (HTT) in the method highlighted in this paragraph. In total, five hybrids with gold content of 0, 1, 3, 5, and 7%, respectively, were obtained. To each of five 100 cm^3^ beakers, 1 cm^3^ of the CNCs sol (*m*(CNCs) = 0.0378 g) and 10 cm^3^ of deionized water were added and then stirred (500 rpm) for 5 minutes. The concentration of CNCs was 0.00063 g cm^−3^. Increasing the stirring speed to 1000 rpm, the pre-calculated amounts of AuNPs were added to the beakers. The solution in each glass was made up to the required volume (which is 80% of the total volume of the autoclave) using (up to 60 cm^3^) deionized water. The hybrid sols were stirred for 30 minutes, placed in an autoclave then subjected to hydrothermal treatment in an oven at 140 °C for 12 hours. The synthesis temperature of Au@CNC was chosen due to the stability of the pure CNCs under hydrothermal treatment conditions.

The appropriate Au@CNC hybrid solution was modified with fluorescent dye rhodamine B to create a system which is humidity sensitive, indicated *via* a change in fluorescence intensity. A hybrid solution was obtained as described by hydrothermal synthesis. To a 100 cm^3^ beaker, 8 cm^3^ of the CNCs sol (*m*(CNCs) = 0.0140 g) and 7.85 cm^3^ of deionized water were added and stirred for 5 min. Then, the pre-calculated amount of AuNPs was added to the mixture. After 12 hours of hydrothermal treatment in an oven at 140 °C, rhodamine B solution (0.240 cm^3^) with a concentration of 4.2 × 10^−5^ M was added to the hybrid sol.

Humidity sensitive hybrid films were created by the evaporation of aqueous Au@CNC suspensions. Hydrothermally treated sols were poured into Petri dishes and dried for 12 hours in an oven at 60 °C. In the case of the fluorescent films, it was done after addition of the dye.

#### 2.1.3 Characterization

X-ray diffraction patterns of freeze-dried CNCs were obtained using an XRD-6000 Shimadzu diffractometer using CuK_α_ radiation (*λ* = 1.54056 Å) at the anode, and a voltage and current of 30 kV and 30 mA, respectively. The X-ray diffractograms were obtained over an angular range of 2*θ* = 5–40° with 0.05° increments.

Fourier transform infrared (FTIR) spectra of freeze-dried CNCs samples were obtained on a Prestige 21 FTIR spectrometer (Shimadzu, Japan) with a wavenumber range of 4000–400 cm^−1^ with 4 cm^−1^.

Apparent sizes and zeta-potential of particles were determined using a Malvern ZetaSizer Nano ZS instrument (4 mW He/Ne laser, 633 nm) at 25 °C in a DTS1070 disposable capillary cell.

UV-vis spectra were obtained using a Solar PB2201 spectrophotometer at a wavelength range of 400–700 nm in 1 cm path length quartz cuvette at room temperature.

Fluorescence measurements were done using a Cary Eclipse spectrofluorometer. The hybrid film was excited at 360 nm and the emission spectra were recorded between 500 and 700 nm, using 5 nm/5 nm slit width, and each spectrum was an average of three scans. A 1.0 cm path length rectangular quartz cell was used for these studies.

Determination of the *morphology* of cellulose and Au@CNC samples, and *EDX analysis* of the Au@CNCs samples were conducted using a scanning electron microscope (SEM) (VEGA3 TESCAN) equipped with an AZtec Energy/X-act detector.

Atomic force microscopy was carried out using a Ntegra Aura (NT-MDT, Russia) in semi contact mode. HA-HR (NT-MDT, Russia) probes were used with a resonant frequency between 210–250 kHz and a spring constant of 14–20 nm. A drop of the sample sol was placed on a smooth glass substrate and dried at 50 °C for 120 minutes. The measurements were carried out in an air atmosphere at a temperature of 24 °C and a relative humidity of 40%. At least ten scans were made of each sample, each of which included at least 65 000 measurement points (interactions).

Response to changes in humidity of the Au@CNC hybrid films was determined using two different approaches. The first approach is the laser diffraction method. The second approach is based on fluorescence measurements taken using a Cary Eclipse spectrofluorometer. The hybrid and control films were excited at 360 nm and the emission spectra were recorded between 500 and 700 nm, using 5 nm/5 nm slit width, and each spectrum was an average of three scans. A 1.0 cm path length rectangular quartz cell was used for these studies.

## 3. Results and discussion

### 3.1. Characterisation of AuNP, CNC, and hydrothermally treated CNC sols

The AuNPs were found to have a negative zeta-potential of −38.7 mV and a bimodal particle size distribution of 10 nm and 70 nm, with a larger portion of the particles being 10 nm in size (Fig. S1A†). The maximum of the absorption spectrum occurs at 536 nm, which corresponds to an AuNP size of 54.1 nm (Fig. S1B†).^20^

The hydrothermal treatment of cellulose resulted in negligible changes in its chemical composition (Fig. S2A†). A slight decrease in peak intensity was observed at 1730 cm^−1^ and 1600 cm^−1^, which may be due to the removal of acetyl groups from the CNC surface (their presence is as a result of the CNC synthesis method used). Fig. S2B† shows uniformity between untreated CNC and CNCs treated at different temperatures, indicating that CNC crystallinity was preserved, and particle structure was maintained despite treatment at such elevated temperatures. High HTT temperatures did lead to a decrease in sol pH, however (Fig. S3†). This can be attributed to the removal of acetic acid residue from the surface of the cellulose nanocrystals to the solution. It is worth noting that our proposed CNC preparation method allows the resulting nanoparticles to be processed under more stringent conditions than CNCs obtained using the more widely known sulfuric acid hydrolysis method. Most notably, unlike with sulfonated CNCs, our CNC particles managed to retain their crystallinity at 150 °C despite being charred.^21^ Therefore, the maximum processing temperature under the indicated hydrothermal conditions at which the stability of the CNCs remains unaffected is 140 °C.

The apparent size of the starting CNC was determined to be 289 nm. Hydrothermal treatment at 120 °C and 140 °C insignificantly affected CNC particle size (Fig. S4A†), but a gradual decrease in their zeta-potential was observed (Fig. S4B†). At processing temperatures of 160 °C and 180 °C, the charge dipped below −20 mV (Fig. S4B†) and the particles lost colloidal stability and coagulated, forming large aggregates (Fig. S4A†).

### 3.2. Au@CNC hybrid nanoparticle characterisation

**Fig. 1:**
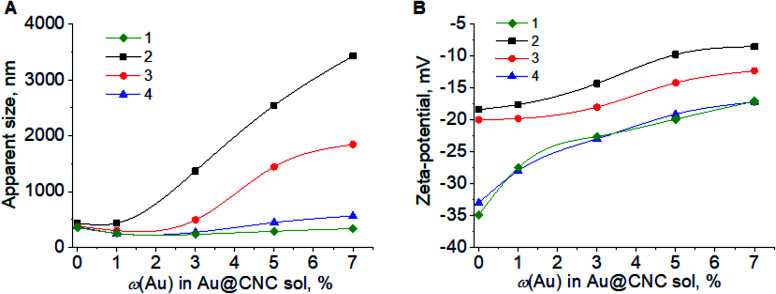
Dependence of apparent size (A) and zeta-potential (B) for Au@CNC sol on the Au mass%: before HTT (1); after HTT (2); 2-fold dilution after HTT (3); and 5-fold dilution after HTT (4).

Hydrothermal treatment of Au@CNC sols led to slight decrease in pH, *ca.* 3–4% (Fig. S5†). The particle size of the hybrid nanoparticles was mostly due to the size of the cellulose nanocrystals (Fig. 1A). With regards to the hybrids obtained after HTT, the particle size notably increased, indicating particle aggregation. Gradual dilution of the sol leads to a decrease in aggregate size and, at 5-fold dilution, the size decreased to that of the cellulose nanoparticles. This nature of coagulation indicates its reversibility and can be attributed to the decrease in the concentration of the background electrolyte upon dilution.

The *ζ*-potential of the sol particles gradually decreased with increasing compositional gold, both before and after HTT (Fig. 1B). However, in the case of the Au@CNC hybrids with gold contents of 5 and 7%, the particle charge became critically small (−8 mV, −9 mV), leading to particle coagulation and sedimentation. In the case of the hybrids with *ω*(Au) = 1–3%, the stability was much higher. However, after 1 day for hybrids with *ω*(Au) = 3%, and 4 days for samples with *ω*(Au) = 1%, the coagulation and sedimentation of particles were also observed (Fig. S6†).

**Fig. 2:**
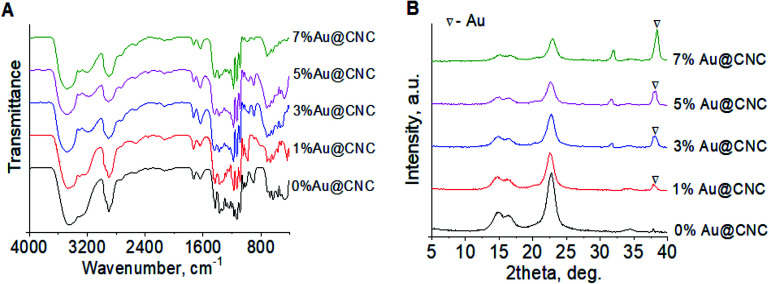
FTIR spectra (A) and XRD patterns (B) of Au@CNC hybrids after HTT.

FTIR spectra of the hybrid samples after HTT showed that the samples' chemical composition remained almost unchanged (Fig. 2A). Most of the peaks related to cellulose are maintained. Therefore, it can be said that the AuNPs interact with cellulose mostly electrostatically and the addition of gold lacks destructive effects on CNC. After HTT, the XRD patterns of the hybrid samples showed peaks related to both cellulose and gold (Fig. 2B).

**Fig. 3:**
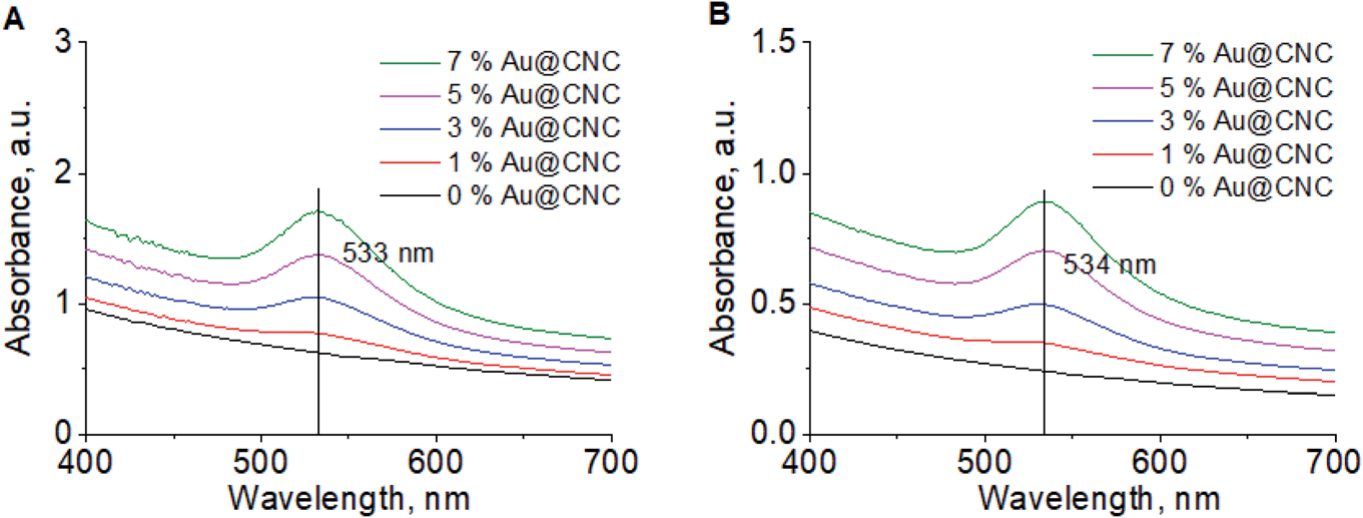
UV-Vis spectra of Au@CNC particles at different Au/CNCs ratios before HTT (A); and after HTT 2-fold dilution (B).

The specific conductivity of the sols is completely dependent on the presence of ions (Na^+^, CH_3_COO^−^, Cl^−^) in solution and corresponds to the conductivity of the supernatant (Fig. S7†). The conductivity was lower prior to HTT due to cleavage of the residual acetate groups from the CNCs surface. Acetyl group peaks were also present (*λ* = 270 nm) in the UV-Vis spectra after HTT (Fig. 3B). *λ*_max_ was found to lie between 520–560 nm on the absorption spectrum, which is characteristic of gold nanoparticles. *λ*_max_ remains the same before (Fig. 3A) and after HTT, indicating that nanogold particle size is preserved. Therefore, it can be concluded that nanocellulose greatly enhances the stability of the gold nanoparticles.

### 3.3. Au@CNC hybrid thin film preparation

Several parameters were considered when deciding which Au@CNC hybrid would be most suitable for humidity sensing. We examined their relative stability, their closeness in structure to pure untreated cellulose, and their gold content.

As previously demonstrated, the 5% and 7% AuNPs Au@CNC colloids were very unstable and particle sedimentation was observed mere hours after HTT (Fig. S6†). The large particle size suggests particle aggregation, which is highly undesirable, as that leads to decreased catalytic activity. The 1% and 3% AuNPs Au@CNC colloids were much more stable.

**Fig. 4:**
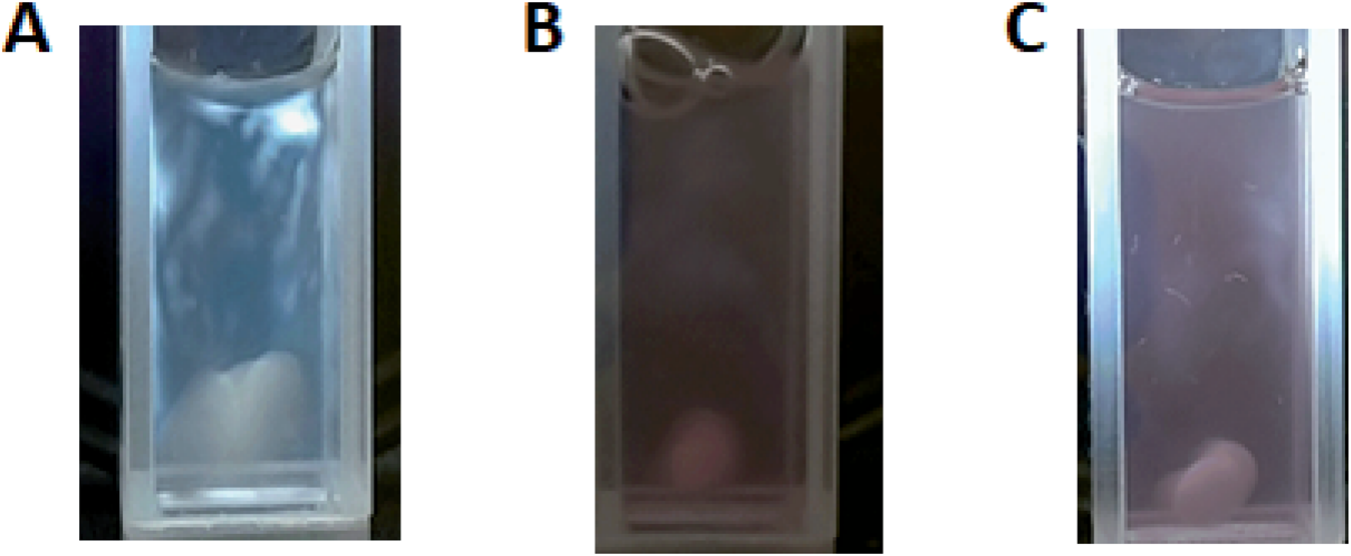
Light birefringence effect of CNC before HTT (A); and 1% AuNPs (B) and 3% AuNPs (C) Au@CNC samples after HTT.

With regards to closeness in structure to pure untreated cellulose, the crystallinity index (CI) of each hybrid was considered. The XRD patterns for the hybrid systems were used to determine their corresponding crystallinity indices. Using Segal's method, the CIs were calculated to be 0.84, 0.81, 0.78, and 0.75 for 1%, 3%, 5%, and 7% AuNPs Au@CNC, respectively. The pure untreated cellulose was found to have a CI of 0.86. The CI values show that as AuNPs content increases, the degree of order decreases. Fig. 4 shows the light birefringence effect for untreated cellulose and 1% and 3% AuNPs Au@CNC sols, confirming their high degree of crystallinity being similar to that of untreated cellulose nanocrystals.

**Fig. 5:**
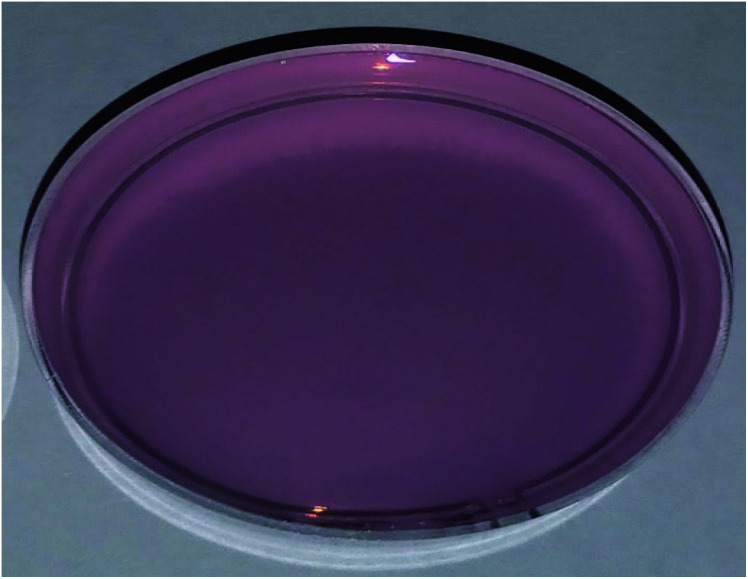
3% AuNPs Au@CNC thin film.

The gold content also played a part when determining which hybrid to use because it has to be high enough to make an appreciable impact on humidity sensing. All factors considered, the 3% AuNPs Au@CNC hybrid is most suitable for humidity sensing and was used to make the thin film. Fig. 5 below shows the 3% AuNPs Au@CNC hybrid thin film obtain from casting.

### 3.4. Structural characterization of hybrid film

**Fig. 6:**
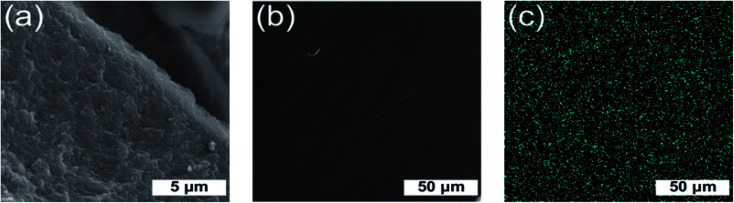
SEM images of Au@CNC hybrid film inner structure (a), surface (b). EDX analysis of the hybrid Au@CNC thin film showing the uniform distribution of gold nanoparticles (blue dots) throughout (c).

Fig. 6 shows that the resulting hybrid film has a lamellar two-dimensional morphology (a) with a uniform distribution of gold nanoparticles (b) and (c) throughout the cellulose matrix. There are no pores, which is expected, due to the formation of a hydrogel with a highly developed morphology during the drying process but, because of capillary pressure and pore collapse, the high developed morphology of hydrogel destroyed.^22^

**Fig. 7:**
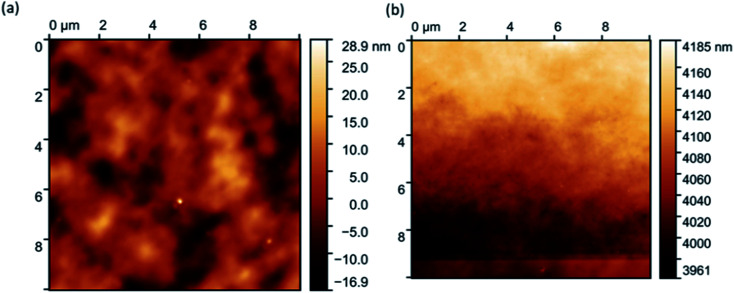
Initial blurry (a) and a clearer image (b) produced after several attempts using AFM.

AFM analysis of the hybrids proved difficult. However, eventual images showed very few (only two) flecks of aggregated gold (Fig. 7a), confirming hybrid stability. Initial images were extremely blurry (Fig. 7a), and several attempts had to be made before the tip of the microscope came into close enough proximity to produce clearer images (Fig. 7b). This is because the hybrid films had a thin layer of water atop them, suggesting that they are hygroscopic.

### 3.5. Au@CNC humidity sensing

**Fig. 8:**
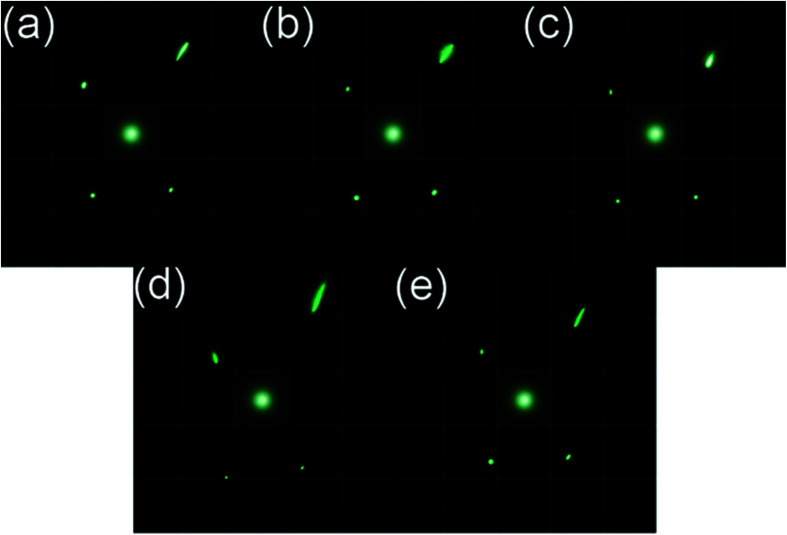
Au@CNC film laser diffraction test at 0 min (a); 1 min into 90% humidity treatment (b); 2 min into 90% humidity treatment (c); 1 min after humidity treatment (d); and 2 min after humidity treatment (e). The size of each square was 17 cm × 17 cm.

We hypothesised that hybrid Au@CNC films respond to changes in humidity. Au@CNC hybrid film response to changes in humidity was determined using two different approaches: laser diffraction method and fluorescence measurements. For the first approach, laser diffraction was carried out using the method described by Ilatovskii *et al.*^23^ Ambient humidity was gradually increased to 90% and then gradually lowered to ascertain how the Au@CNC film responded to changes in humidity. As can be seen from Fig. 8, the film is holographic and undergoes changes in its refractive index due to the adsorption/desorption of ambient moisture and because of CNC's high surface area.^24,25^ The diffraction pattern changes as humidity increases before relaxing back to its initial state as ambient moisture decreases back to the starting humidity, strongly indicating reversibility (Fig. 8 and S8†). Not only does the film respond to changes in ambient humidity, it also does so quickly and autonomously, returning to its initial state within 2 minutes and without the need for drying. This makes it an ideal candidate for real-time humidity sensing.

The second approach is based on fluorescence intensity measurements. This method was used to determine the role of AuNPs in humidity sensing. Water molecules are effective fluorescence quenchers.^26–28^ Several publications have also shown AuNPs to quench fluorescence.^29–31^ It was assumed that the hybrid film would be more effective at fluorescence quenching than the control. To test this, fluorescent rhodamine B dye was added to the sols (hybrid and untreated CNC) before casting films. The films were then exposed to different levels of humidity (up to 80%) and the relative decrease in fluorescence intensity calculated. Fluorescence quenching was determined using the equation below:
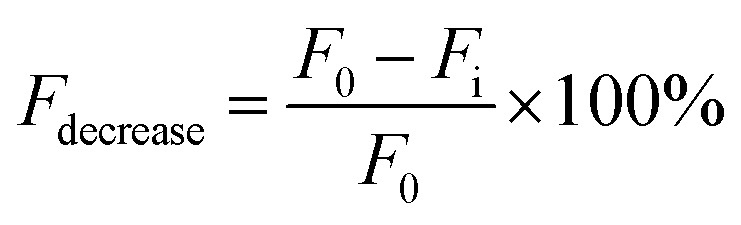
where *F*_0_ – measured film fluorescence at ambient humidity and *F*_i_ – measured film fluorescence at a humidity different from ambient.

**Fig. 9:**
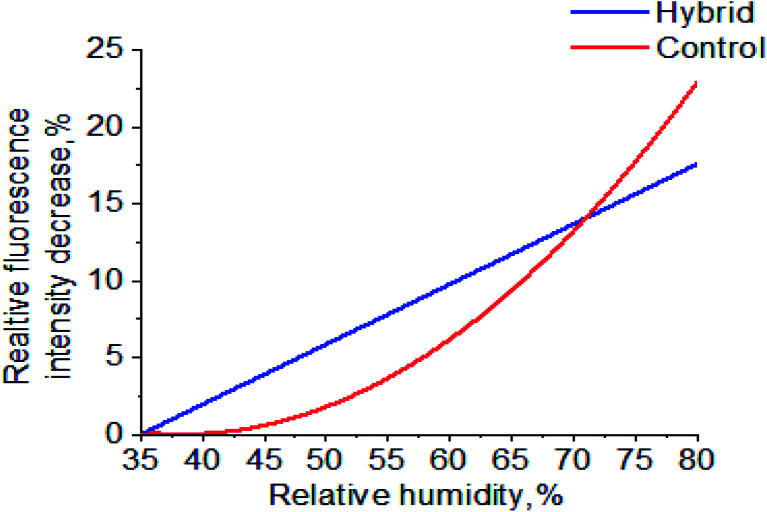
Au@CNC film doped with rhodamine B fluorescence quenching test at ambient humidity up to 80% humidity.

As Fig. 9 shows, the sorption of water molecules in both the rhodamine B-doped hybrid and control films affects dye fluorescence, causing a decrease in intensity to be seen. However, a more drastic decrease is observed between 35% and 70% humidity for the hybrid film. This can be attributed to the AuNPs dispersed throughout the matrix. It was assumed that, for both cases, the mechanism is based on the Förster resonance energy transfer (FRET) phenomenon, where donors (excited dye molecule) transfer their energy to acceptors (water molecules or AuNPs) through non-radiative dipole–dipole interactions.^32^ The decrease in fluorescence intensity for the control shows a polynomial dependence on change in humidity. Contrarily, the hybrid film shows a linear dependence, a desirable property when it comes to sensors as such sensors are easier to control and highly sensitive systems can be created based on them. The doped Au@CNC film can be used as an indicator of moisture content as fluorescence intensity is dependent on it.

## 4. Conclusion

To conclude, we proposed a simple green synthesis method for preparing hybrid Au@CNC nanoparticles using hydrothermal treatment. Characterisation of the environmentally friendly hybrid Au@CNC sol showed that hybridisation was successful, and that CNCs greatly enhance AuNP stability by minimising homoaggregation. Hybrid Au@CNC films are hygroscopic and respond to changes in humidity. The cellulose matrix is active, autonomously contracting and relaxing with the adsorption and desorption of water molecules in real-time. AuNPs enhance hybrid response to ambient moisture and show a linear dependence on changes in humidity, making the hybrid controllable, highly sensitive, and a viable prospective material for humidity sensing applications. The main interest according to the creation of humidity sensitive materials in terms of Au@CNC hybrids is to determine the most suitable structure for industrial production and everyday application.

The rhodamine B-doped AuNP/CNC hybrid sensor can find real world applications such as smart packaging in various fields. The degree of intensity of rhodamine B dye fluorescence can be used as an indicator of moisture content. This is particularly helpful in industries where the quality of goods is dependent on the amount of moisture present – such as in the food industry where high moisture content can be indicative of food spoilage.

## Conflicts of interest

The authors confirm that there are no conflicts of interest as no financial support for this work has influenced our findings.

## Supplementary Material

RA-010-D0RA07300H-s001
