# Supplementary material for: Environmentally friendly Au@CNC hybrid systems as prospective humidity sensors
Source: RSC Adv. 2020 Sep 21;10(58):35031–8. doi: 10.1039/d0ra07300h (PMC9056851; doi:10.1039/d0ra07300h)
Supplement: RA-010-D0RA07300H-s001 [file RA-010-D0RA07300H-s001.pdf]

Electronic Supplementary Material (ESI) for RSC Advances.
This journal is © The Royal Society of Chemistry 2020

Environmentally friendly Au@CNC hybrid systems as prospective humidity sensors

Electronic Supplementary Information

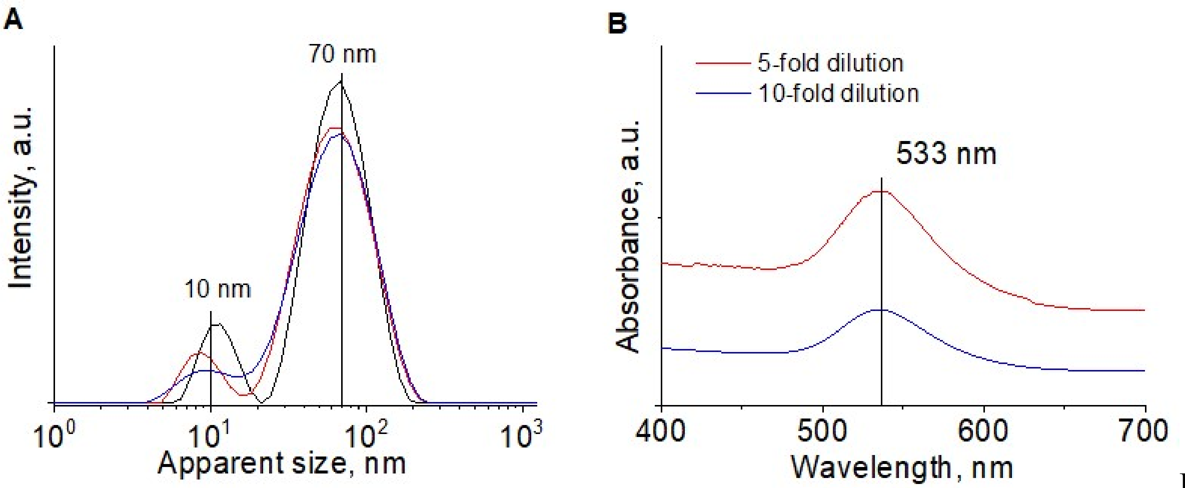

Figure S1. Size distribution of AuNPs obtained using DLS (A) and UV-vis spectra of AuNPs sols (B).

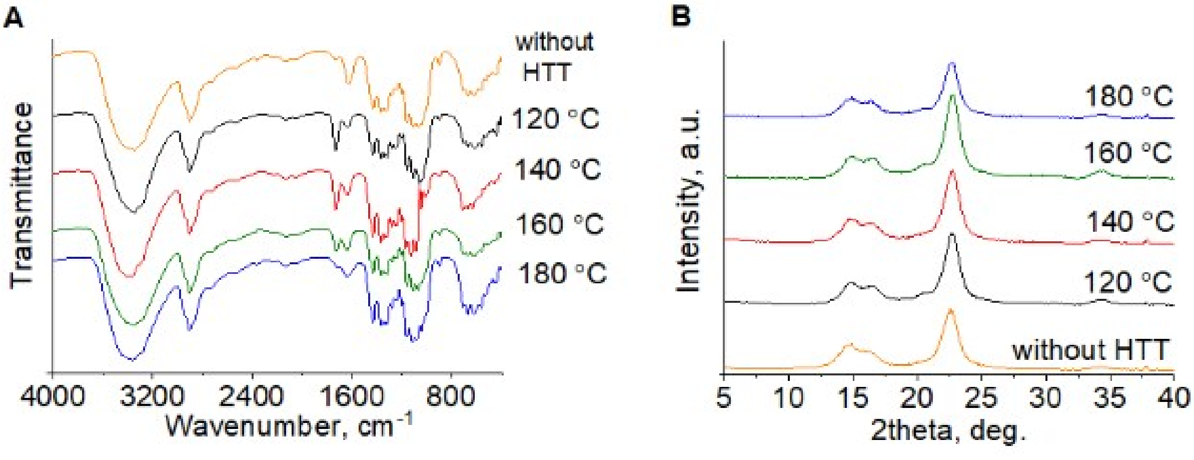

Figure S2. FTIR spectra (A) and XRD patterns (B) of CNCs samples before and after HTT.

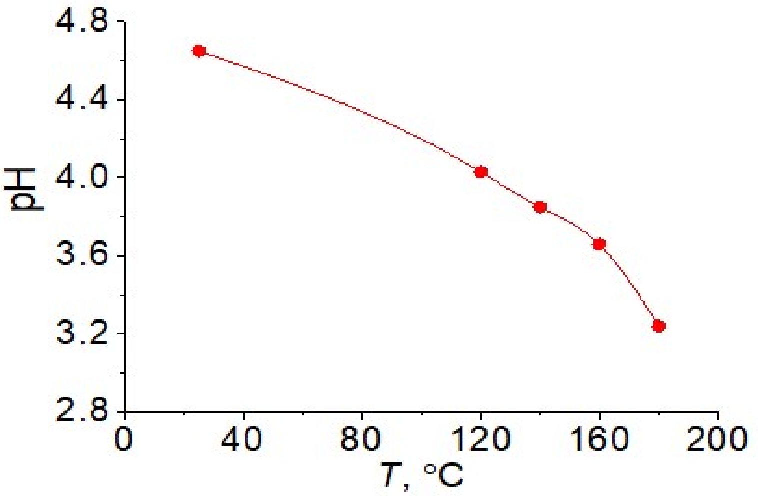

Figure S3. pH dependence of CNC sol on HTT temperature.

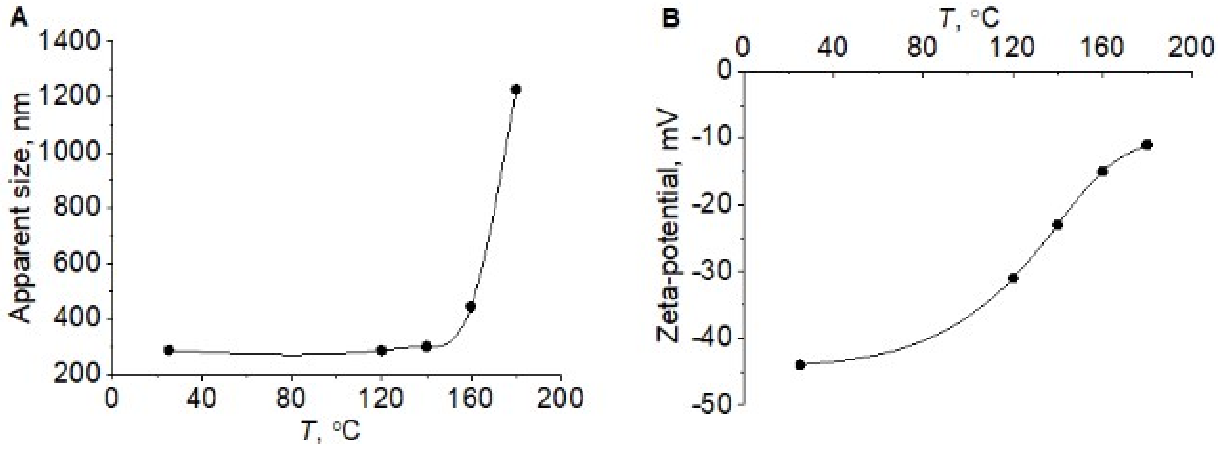

Figure S4. Dependence of apparent size (A) and zeta-potential (B) for CNCs on the temperature of HTT.

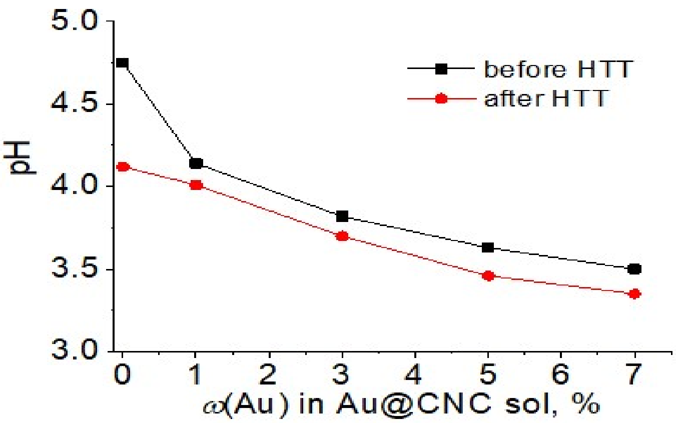

Figure S5. Dependence of pH value on gold content in Au@CNC hybrid sol before and after HTT.

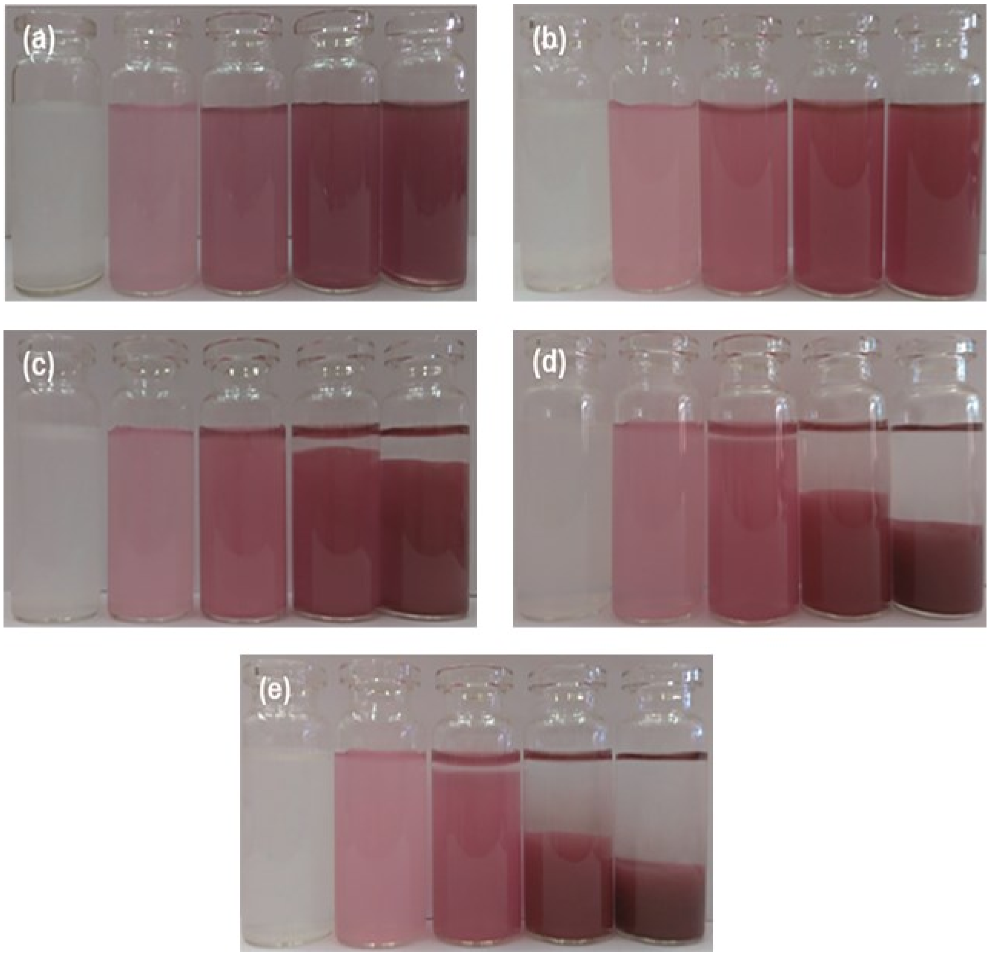

Figure S6. Digital images of CNCs sols with 0, 1, 3, 5, 7 mass % Au NPs before HTT (a); and Au@CNCs hybrid NPs after HTT (b); after 10 min undisturbed (c); after 4 h undisturbed (d); after 4 days undisturbed (e).

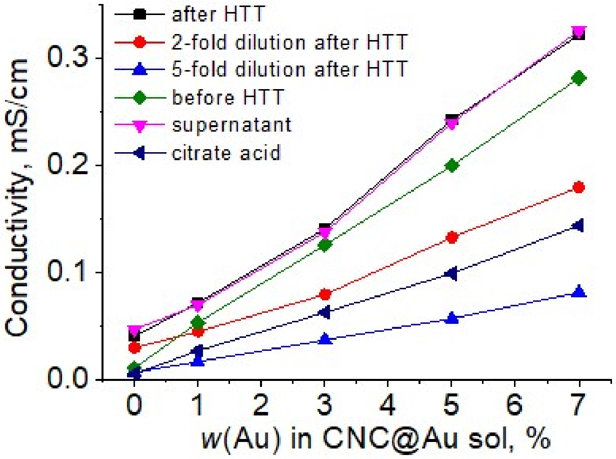

Figure S7. Conductivity of Au@CNCs sols: before HTT (1); after HTT (2); 2-fold dilution after HTT (3); 5-fold dilution after HTT (4); supernatant (5); and citric acid (6).

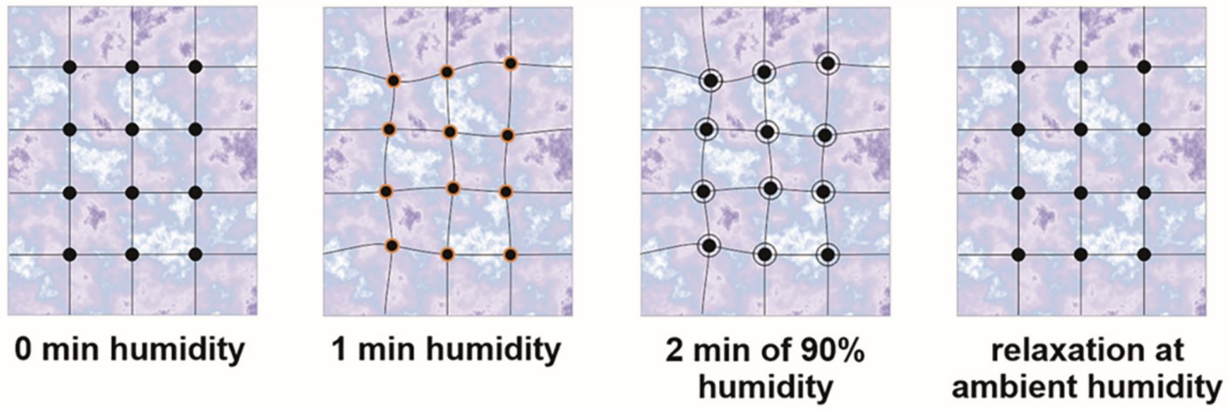

Figure S8. Au@CNC film diffraction pattern responding to changes in humidity.
